# Report of the Committee on Hygiene1Read at the ninety-sixth annual meeting at Albany, January 28, 1902.

**Published:** 1902-03

**Authors:** Henry Reed Hopkins, Geo. B. Fowler, J. H. Pryor, J. M. Mosher, M. A. Veeder, J. L. Heffron

**Affiliations:** Chairman


### Report of the Committee on Hygiene.[1]

BY HENRY REED HOPKINS, Chairman, and Colleagues.

*To the Medical Society of the State of New York:*

THE prominent topics emphasised in the recent reports of your committee, continue to be the subjects demanding the attention of the sanitarian.

The enormous death-rate from typhoid fever in the state attracts the attention of all well informed persons to the con-

1. Read at the ninety-sixth annual meeting at Albany, January 28, 1902.

tinued impurity of the water supply of the entire state, to the lack of intelligence and appreciation of this prime necessity of health, to the corresponding want of efficiency as hygienists of our medical profession.

That there is more than four times the morbility and mortality from typhoid fever, in the chief cities of this state, than in the large cities of Europe, is a most humiliating demonstration.

Your committee again directs attention to the fact that in the main the responsibility for this disgraceful state of impure water supply, and the consequent loss of time, money, and life from typhoid fever rests upon the medical profession as the same is represented in the Medical Society of the State of New York, and calls upon the several local societies to make this question one of especial study to the end that those in authority may speedily be induced to apply the proper remedy.

The real sanatory problem of the day—than which there has been none more important in history, the prevention and cure of consumption by state control—has made slow but positive progress. The tactics of the enemies of the cause seem to have been to confuse, to distract, to delay, to obstruct by impossible suggestions of help. A state sanatorium for the treatment of cases of incipient consumption was demanded by abundant and conclusive experience, advised by this society and this demand was supported by overwhelming public sentiment. The selection of a site furnished the occasion for meddlesome and mischievous suggestions, an artificial activity of some self-styled friends of the cause, and required much patience, wisdom and loyalty on the part of the right-minded members of the board of trustees, in order that a wrong choice might not ruin the enterprise at its beginning. The delay thus caused has been most exasperating, and trying, but has given opportunity of testing how sane the movement is, how imperative is the demand on the part of the people, and of the futility of attempting to kill that which is demanded in the interests of the public health.

Your committee with all emphasis renew and reaffirm what it has heretofore declared as to the importance of this matter, and advise that this society renew its pledge of confidence and support to the cause of state promotion and cure of consumption in sanatoria as the state's highest duty and most glorious opportunity.

Your committee has given consideration to the recent increase of knowledge regarding the relation of some of the smaller insects to the spread of disease. The rôle of certain varieties of mosquitoes to the spread of malaria and yellow fever opens a vista for the prevention of these diseases of immense attractiveness and potentiality. It would seem that the near future is to demonstrate that some of the fairest portions of the earth, that have hitherto been thought unsuitable for Anglo-Saxon inhabitation, by reason of malarial endemicity are to be reclaimed, and that modern sanitary science is not only to drive yellow fever from off the earth as it has already been driven from Cuba and Porto Rico, but that by a knowledge of the ague-bearing mosquito, the sphere for Anglo-Saxon residence and activity is to be enormously expanded.

The continuous and unusual presence of smallpox in various parts of the state moves your committee to urge upon the society the importance of a more intelligent supervision of the results of vaccination, the importance of greater care and watchfulness in determining that primary vaccinations are successful, in demanding legislation requiring a general revaccination of all persons at puberty and after puberty, at least once in ten years, and that no child be permitted to attend any public or parochial school unless the same can show the marks of thorough vaccination.

Here your committee is minded to refer to, and to deplore and protest the policy of the present administration of the state in reference to the public health and the state board of health. Your committee has the conviction, that it is not good statesmanship to neglect or to minimise the ordinary means found in operation in all civilised communities for the protection of the public health; and that in the whole history of extravagant and wasteful expenditure, there is nothing more extravagant and wasteful, than a penurious economy in appropriations for the prevention of the preventable diseases; that in the department of public health should ever be found men of the highest ideals and the noblest attainments, for the reason that the problems they encounter are the most intricate, trying and important that ever engaged the mind of man, and that it is of prime importance that the men of this department should be leaders of thought and always in full command of the confidence of the people.

How shallow is the intelligence, how thin the veneer of statesmanship, what a bar to real economy and progress is the policy which fixes the annual salary of the state commissioner of health at $3,500 when the commissioner of fisheries and game gets $5,000; the state architect, $7,500, and the three railroad commissioners, $8,000 each? And how humiliating is the easy criticism of the politician, and sometimes law-maker, who asks, what does the state board of health do, and answers himself—Oh, they keep a record of the dead ones, and get well paid for their work!

The practical working of the unfortunate schisms in medicine is seen in destroying the public confidenee even in medical measures for the protection of the peoples lives, by the prevention of the preventable diseases, and shows its real colors in the indifference of our legislators to the appeals or advice of this society upon matters of public health, in the penurious appropriation for the maintenance of our state board of health and in the conspicuously inadequate salary of the state commissioner of health.

The members of the legal profession seem to fight each other vehemently in court, but are not known to quarrel among themselves when urging that the salary of the judge be made commensurate with the dignity of his office, and it is so.

The people have ever reserved to themselves the right to say who shall practise the healing art, and what qualifications if any such persons shall have. The questions of what preliminary education such persons shall have, what shall be considered to constitute the science of preventive medicine, a knowledge of which all persons who practise any part of the healing art shall have, are ones upon which the people will probably listen to, and possibly be advised by this society. The broader questions of how the healing art shall be practised, what the size of a dose of a particular remedy for a particular disease shall be, whether remedies shall be gathered from nature's inorganic kingdom, or from her organic storehouse, or from both sources at will, whether men shall use water within and without, massage, electricity, hypnotism, or mental suggestion, with or without the added enforcement of religious belief, are those concerning which the people have ever shown themselves unwilling to be advised and intolerant of dictation.

Not understanding or not comprehending the spirit of the people in this important direction has in times past placed this society in a false light upon questions of great popular interest, if not of public importance.

Read in the light of history it seems clear that it would have been better for the medical profession, better for the great interests of the public health, which the medical profession must ever guard, if this society could have refrained from seeming to want to dictate to the people how they should be doctored, could have contented itself with advising, and could have withheld that advice until it was requested.

Your committee is compelled to force these observations upon your attention by the logic of recent events. The people of the state are experimenting with osteopathy and Eddyism, just as a few years ago they were experimenting with homeopathy and eclecticism. There is great danger now, as then, that this society wishing to advise from abundant knowledge, will be found to seem to dictate to the people how the people shall conduct their own affairs; that the ever to be lamented blunders of 1857 and 1865 may be repeated in this the beginning of the new century. It would seem that this society should have learned by sad experience, that the right is not always expedient, that it is wiser to be content to advise, and perchance to guide in medical questions, to be content to memorialise and to protest with becoming dignity, remembering that the weightier matters,—higher medical education, increased medical efficiency and better protection to the public health,—may only be adequately promoted and prospered by a united medical profession inspiring, guiding and leading a sympathetic and appreciative people.

H. R. Hopkins, *Chairman*.

Geo. B. Fowler.

J. H. Pryor.

J. M. Mosher.

M. A. Veeder.

J. L. Heffron.

